# The occurrence, progression and development of four types of gastric mucosal atrophic lesions and their histopathological characteristics

**DOI:** 10.1007/s10120-023-01400-6

**Published:** 2023-06-17

**Authors:** Yangkun Wang, Junling Zhou, Nianlong Meng, Binfeng Yang, Chaoya Zhu, Bo Jiang, Sunan Wang, Xiaodong Chen

**Affiliations:** 1 Department of Pathology, Shenzhen Longgang District Fourth People’s Hospital, Shenzhen, 518123 Guangdong China; 2 Department of Pathology, Shenzhen Nanshan District People’s Hospital, Shenzhen, 518055 Guangdong China; 3 Department of Pathology, 989th Hospital of PLA, Luoyang, 471000 Henan China; 4 Department of Pathology, Xinxiang Central Hospital, Xinxiang, 453000 Henan China; 5 Department of Pathology, The Third Affiliated Hospital of Zhengzhou University, Zhengzhou, 450052 Henan China; 6 Department of Pathology, The 990th Hospital of the PLA Joint Logistic Support Force, Zhumadian, 463000 Henan China; 7 Department of Pathology, Shenzhen Vocational and Technical College, Shenzhen, 518110 Guangdong China; 8 Shenzhen Vocational and Technical College, Bank of Xili Lake, Xilihu Town, Nanshan District, Shenzhen City, 518055 China

**Keywords:** Stomach neoplasms, Atrophic lesions, Histomorphology, Pathological staging, Immunohistochemistry

## Abstract

**Objective:**

To investigate the occurrence and development of gastric mucosal atrophic lesions and their histopathological characteristics.

**Methods:**

Histopathological diagnosis and immunohistochemical staining using the EnVision two-step method were conducted on 1969 gastric mucosal atrophic lesions obtained from gastroscopic biopsy specimens. A total of 48-month three-stage endoscopic biopsy follow-ups were performed.

**Results:**

When the gastric mucosal epithelium was affected by infection, chemical irritation, or immune or genetic factors, the gastric mucosal epithelium glands atrophied, the mucosa became thinner, the number of glands decreased, the intestinal epithelium progressed to metaplasia and smooth muscle fibre became hyperplasia. Such changes may lead to the proliferation and dysplasia of epithelial cells of the gastric mucosa and neoplastic hyperplasia in nature; this is referred to as gastric mucosal atrophic lesions in this study. According to this definition, the present study divided gastric mucosal atrophy into four types: (1) glandular atrophy of the lamina propria; (2) compensatory proliferative atrophy; (3) intestinal metaplasia atrophy; and (4) smooth muscle proliferative atrophy. The incidence rates of the above were 40.1% (789/1969), 14.3% (281/1969), 27.8% (547/1969) and 17.9% (352/1969), respectively. One- to 4-year follow-ups found that the changes were not significant and that the percentages of patients with disease exacerbation were 85.7% (1688/1969) and 9.8% (192/1969). The percentages of patients who developed low-grade intraepithelial neoplasia and high-grade intraepithelial neoplasia were 2.8% (55/1969) and 1.1% (21/1969), respectively; 0.7% (13/1969) of patients developed intramucosal cancer.

**Conclusion:**

Gastric mucosal atrophic lesions and histopathological staging are based on the morphological characteristics of gastric mucosal atrophy and the hypothesis of malignant transformation of cells during the occurrence and development of mucosal atrophy. Mastering pathological staging is beneficial to clinicians for enacting precise treatment and is important for reducing the incidence of gastric cancer.

## Introduction

In recent years, with the application of many new gastroscopes, such as virtual endoscopy, magnifying endoscopy and ultrasound endoscopy for clinical screening, the standardisation of the histopathological diagnosis of gastroscopic biopsies has become more important [1–3]. Gastric mucosal atrophy has traditionally been defined as the loss of glands. Despite the simplicity of this definition, histopathologists have encountered considerable difficulty in agreeing on the nature, presence and grading of gastric atrophy [4, 5]. Gastric mucosal atrophy is an important part of gastric mucosal biopsy tissue and is one of the most common lesions found throughout hospitals worldwide [6–8]. Currently, the updated Sydney System proposes the use of a visual analogue scale as a diagnostic criterion for gastric mucosal atrophy and has achieved good reproducibility [9]. It has been reported in the relevant literature that atrophy and non-atrophy of the gastric mucosa, along with the border of mucosal atrophy, can be distinguished by the endoscopic observation of changes in mucosal colour [10, 11]. Atrophy is the least reproducible criterion of the updated Sydney System; however, the histopathological changes observed using haematoxylin and eosin (HE) staining or the others could be more stable and universal, and the classification and grading of gastritis and the distinction between atrophy and non-atrophy are emphasised in histopathology [12]. Most gastric cancers occur in the context of atrophic gastritis, and the risk increases with the degree of atrophy [13]. In addition, there is considerable disagreement among pathologists regarding the definition of gastric atrophy and its severity. Gastric mucosal atrophy has not been completely distinguished from atrophic gastritis. Chronic atrophic gastritis is classified into three grades: (1) mild atrophic gastritis, which is characterised by a reduction in the number of intrinsic glands by 1/3 of the original glands; (2) moderate atrophic gastritis, which is characterised by a reduction in the number of intrinsic glands between 1/3 and 2/3 of the original glands; and (3) severe atrophic gastritis, which is characterised by a reduction in the number of intrinsic glands by more than 2/3 of the original glands [14–17]. However, this classification has some shortcomings. Atrophy can occur in the intestinal type of gastric cancer as well as in the gastric type. The previous study conducted by the authors of the present study reported that *Helicobacter pylori* infection leads to gastric small concave epithelial type signet-ring cell carcinoma [18]. In severe atrophy of the lamina propria glands, there is no cellular heterotypic hyperplasia or intestinal metaplasia, including complete intestinal metaplasia and incomplete intestinal metaplasia. Complete intestinal metaplasia is defined as the presence of goblet cells without acidic alcian blue or periodic acid–Schiff positive material in columnar-type cells. Incomplete intestinal metaplasia is defined as the presence of goblet cells with acidic mucins in the goblet and adjacent columnar-appearing cells. The atrophy of the gastric mucosal glands is not always accompanied by inflammatory cell infiltration, i.e. gland atrophy is not always caused by chronic inflammation. Infection of the gastric mucosa, long-term drug stimulation and immune and genetic factors can lead to atrophy of the gastric mucosa. In the compensatory proliferation period of gastric mucosa atrophy, the gastric mucosa is not thinned but thickened; therefore, the thickness of the mucosa cannot be used as a diagnostic criterion for gastric mucosa atrophy. This study aims to investigate the occurrence and development of gastric mucosal atrophic conditions and their histopathological characteristics.

A total of 1,969 gastric mucosal biopsy samples and follow-up patients were chosen from the pathology departments of six hospitals. Four types of gastric mucosal atrophic lesions were proposed according to their pathological stages and characteristics as follows: (1) stage I gastric mucosa gland atrophy: mild atrophy and a smaller volume of the gastric mucosa glands; (2) stage II compensatory proliferative atrophy: polar compensatory hyperplasia and reduced gastric mucosa glands; (3) stage III intestinal metaplastic atrophy (intestinal metaplasia is a transformation of the cells in the lining of the upper digestive tract, often the stomach): a dominant intestinal metaplasia type and the intestinal metaplasia cells accounted for > 30% of the entire gland; and (4) stage IV smooth muscle proliferative atrophy: proliferative atrophy of smooth muscle fragments and smooth muscle fibres of different sizes and shapes appeared in the lamina propria. These four types of classifications are based on a previous study [18]. Mastering the histological characteristics and pathological stages of atrophic conditions is beneficial to clinicians for the precise treatment of atrophic lesions of the gastric mucosa and the tracking of malignant cell transformation, which is important for reducing the occurrence and development of gastric cancer.

## Materials and methods

### Research participants

This study represents prospective research. A total of 1969 patients with gastric mucosal atrophic lesions were diagnosed using gastroscopic biopsy histopathology between July 2017 and July 2021, including patients with atypical hyperplasia. Information about the causes of the atrophic lesions was collected from the diagnosis records of the aforementioned hospitals. The patients comprised 1227 men and 742 women. There were 372 patients with gastric angle, 979 patients with gastric antrum, 476 patients with a gastric body, 125 patients with gastric fundus and 17 patients with cardiac atrophies. For the tissue sampling method, all the tissue samples included in this study were collected from patients using the biopsy protocol; the protocol for all patients kept strong consistency. Three-to-five pieces of mucosal tissue were clamped from each site, fixed in 10% neutral formalin, routinely dehydrated, embedded in paraffin, sectioned at a thickness of 4 μm and stained by HE and immunohistochemistry. This study was approved by the Ethics Committee of Foresea Life Insurance Guangzhou General Hospital.

### Immunohistochemical staining

Using the EnVision two-step method, tissue sections were deparaffinised, hydrated and rinsed with distilled water. The sections were then placed in tris-buffered saline (TBS) for 10 min. Endogenous peroxidase was then blocked for 5 min, followed by incubation with TBS for 10 min. Each primary antibody was purchased from Thermo Fisher Scientific Inc. (Waltham, Massachusetts, US), Mucin 5AC (MUC5AC), Mucin6 (MUC6), Mucin2 (MUC2), spinal muscular atrophy (SMA), desmin (DES), villin, caudal type homeobox 2 (CDX2), p53, Ki-67) and incubated with the sections for 30 min at room temperature (25℃). After washing in TBS and incubation in EnVisionTM, incubation with a second antibody was performed for 10 min. The chromogenic substrate solution was used before being rinsed with distilled water. The sections were developed with 3,3′-diaminobenzidine (DAB) and re-stained with haematoxylin. Known sections of the gastric mucosa were used as positive controls, while a phosphate-buffered saline (PBS) buffer was used as a negative control. The working solutions were purchased from Fuzhou Maixin Biotechnology Development Co., Ltd, China, and the operational steps were performed strictly according to the kit’s instructions.

### Follow-up visits

In this study, 1969 patients who met the study requirements of this group were followed up with from July 2017 to July 2021 (a total of 48 months), with a follow-up deadline of July 31, 2021. The follow-up was conducted by an endoscopic biopsy to observe the progression of gastric mucosal atrophic conditions, and the biopsy review was divided into three phases: (1) 1–3 months; (2) 4–6 months; and (3) 7–12 months. The follow-up of the biopsy gastric mucosa review was conducted six times at the most and two times at the least. Regular re-biopsy was conducted due to the long period between July 2017 and July 2021, and most patients with atrophic lesions of the gastric mucosa were progressively aggravated and were needed for the follow-up of malignant cell transformation of atrophic lesions of the gastric mucosa.

### Statistical analysis

The SPSS 22.0 software was used for statistical analysis in this study. Gender, age and each type of statistical method were tested using the Chi-square test, and results of *P* < 0.05 were considered statistically significant.

## Results

### Clinical characteristics

Gastric mucosal atrophic conditions were found in 1227 men and 742 women, with a ratio of 1.65:1. There were more men than women in all pathological histological stages. Among the patients, 61.1% (1204/1969) were aged ≤ 60 years at onset, and 38.9% (765/1969) were aged > 60 years at onset; the percentage of patients aged ≤ 60 years at onset was higher than patients aged > 60 years at onset (Table 1).

**Table 1:** Staging and histopathological features of atrophic lesions of gastric mucosa

Types	Histological characteristics	Immunophenotype
Stage I, lamina propria gland atrophy		
Stage IA, mild atrophy of the lamina propriae glands	The proliferative zone in the deep gastric pits, the canyons and the neck of the gastric gland was structurally disorganised or absent, with increased interstitium, smaller glands, and wider glandular intervals. There were 0–2 mitotic figures/HPF in the proliferative zone region. The number of glands in the lamina propria was reduced to 1/2 of the original glands, the surface epithelium was also reduced, and the gastric pits were shallower	The number of ki67-positive cells in the proliferative zone accounted for 3–11%. The number of MUC5AC and MUC6-positive cells decreased unequally
Stage IB, severe atrophy of lamina propria glands	The proliferative zone in the deep gastric pits, the canyons and the neck of the gastric glands was structurally disordered or absent, with increased interstitium, smaller and fewer glands, and more pronounced widening of the glandular intervals. There were 0–5 mitotic figures/HPF. The number of glands in the lamina propria was reduced to more than 1/2 of the original number of glands. The surface epithelium was also reduced at the same time, and the gastric pits were shallower	The number of ki67-positive cells in the proliferative zone accounted for 3–15%. MUC6-positive cells decreased or disappeared; MUC5AC-positive cells decreased
Stage II, compensatory proliferative atrophy		
Stage IIA, polar compensatory hyperplasia	The decrease in the pyloric/fundic glands was accompanied by a significant proliferation of stem cells and cervical mucus cells in the proliferative zone; the surface epithelial cells proliferated in a characteristic papillary pattern, usually reaching 0.3–0.6 mm in height, with the proliferating glands and cells still maintaining normal polarity	Ki67-positive cells accounted for 5–10% of the compensatory hyperplastic areas. MUC5AC-positive cells increased; MUC6-positive cells decreased or disappeared
Stage IIB, disordered compensatory hyperplasia	Decreased pyloric/fundic glands, significant proliferation of stem cells and cervical mucus cells in the proliferative zone were more evident, and surface epithelial cells still proliferated in a characteristic papillary pattern, usually reaching a height of 0.5–1.2 mm. At the same time, there was a marked disturbance of proliferation polarity, with glands of surface epithelial cells and cervical mucus cells appearing in the region of the pyloric/basal gland, a morphological feature of this stage	Ki67-positive cells accounted for 10 to 19% of the compensatory hyperplastic areas. MUC5AC-positive cells increased; MUC6-positive cells decreased or disappeared
Stage III, intestinal metaplasia atrophy		
Stage IIIA, dominant intestinal metaplasia	Intestinal metaplasia cells accounted for more than 30% of the entire gland, and intestinal metaplasia areas were more than three consecutive gastric areas (2–6 mm in one gastric area), or multiple intestinal metaplasia lesions appeared. The cells of intestinal metaplasia were mildly hyperplastic without atypia. The glands in the pyloric/fundic glands became smaller and decreased in number	Ki67-positive cells accounted for 5–15% of the intestinal metaplasia areas. Positive MUC2 expression
Stage IIIB, antagonistic intestinal metaplasia	The extent of intestinal metaplasia was the same as that of dominant intestinal metaplasia. Histological features showed significant antagonistic proliferation, mainly manifested as atypical proliferative cells on one or both sides of intestinal metaplasia cells, or atypical proliferative glands in the surrounding glands of intestinal metaplasia glands, that is, intestinal metaplasia antagonistic. Pyloric glands/fundic glands were markedly reduced or absent	Ki67-positive cells accounted for 15–25% of the antiproliferative regions. Positive MUC2 expression
Stage IV, smooth muscle hyperplastic atrophy		
Stage IVA, smooth muscle lamellar hyperplasia	Smooth muscle fibres of various sizes and morphologies were present in the lamina propria and separated to surround the pyloric/fundic glands. Smooth muscle at the base of the lamina propria showed bundles deep into the lamina propria, separating the lamina propria glands into nests of cells of varying sizes. Stem cells and cervical mucous cells in the gastric proliferative zone had different degrees of compensatory hyperplasia and intestinal metaplasia	Ki67-positive cells accounted for 10–25% of the hyperplastic areas. SMA and DES were positively expressed
Stage IVB, smooth muscle fibre plate hyperplasia	There was significant smooth muscle hyperplasia in the lamina propria, and the hyperplastic smooth muscle extended from the mucosal muscle layer to the mucosal proliferative zone in severe cases, and the hyperplastic smooth muscle together with the mucosal muscle layer formed a characteristic myofibrillar plate structure. Stem cells and cervical mucous cells in the gastric proliferative zone had different degrees of compensatory hyperplasia and intestinal metaplasia	Ki67-positive cells accounted for 20–25% of the hyperplastic areas. SMA and DES were positively expressed
Intraepithelial neoplasia		
Low-grade intraepithelial neoplasia	Epithelial cells showed mild to moderate atypia, with elongated but still polar nuclei located at the base of the glandular epithelium, and small to medium-sized nucleoli were visible. Low-grade neoplasia occupied part or all of the gastric mucosa. Mitotic figures ranged from 2 to 4/HPF	ki67-positive cells accounted for 30–50%. CDX2, villin, and p53 were positively expressed
High-grade intraepithelial neoplasia	The atypia of epithelial cells was obvious, the cells changed from columnar to cuboidal, the nuclei were large, the nucleoplasmic ratio was increased, the nucleoli were obvious, and the mitotic figures were increased. High-grade neoplasia occupied almost the entire layer of the gastric mucosa. Enlarged and distinct nucleoli were observed in about 30%–50%. Mitotic figures ranged from 3 to 6/HPF	ki67-positive cells accounted for 30–50%. CDX2, villin, and p53 were positively expressed
Intramucosal adenocarcinoma	The cancerous tissue formed glandular ductal papillae and occupied the entire layer of the gastric mucosa. Cytologically, the cells had large nuclei, an increased nucleoplasm ratio, and enlarged and distinct nucleoli visible in ≥ 50% of the nuclei. Mitotic figures ranged from 3 to 8/HPF	ki67-positive cells accounted for 30–50%. CDX2, villin, and p53 were positively expressed

*HPF* high power fields. *MUC5AC* mucin 5AC, *MUC6* mucin6, *CDX2* caudal type homeobox 2, *SMA* spinal muscular atrophy, *DES* desmin

### Schematic diagram of the occurrence and development of gastric mucosal atrophic conditions

**Fig. 1:**
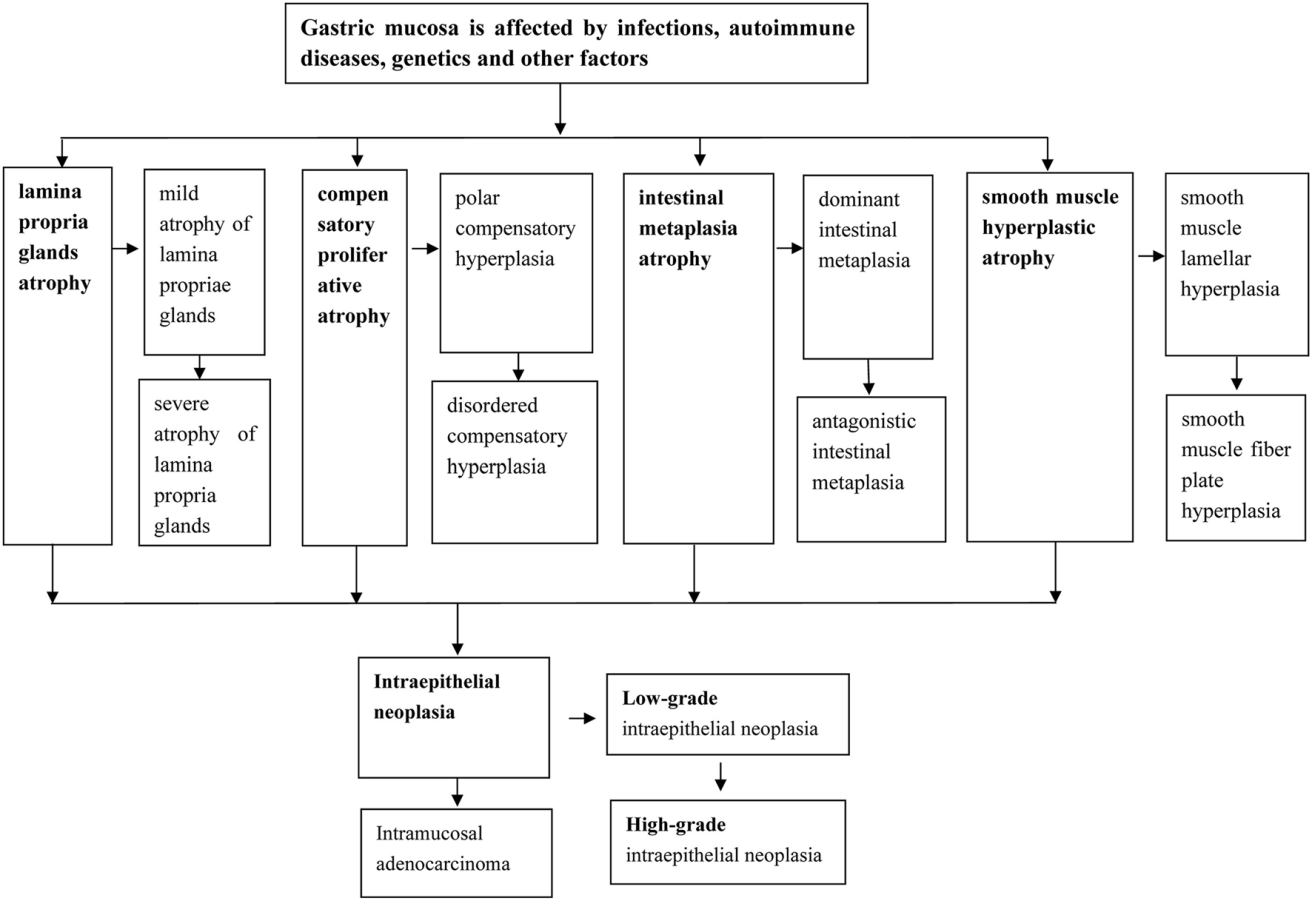
Schematic diagram of the classification of the occurrence and development of atrophic gastric mucosal lesions

Infection of the gastric mucosa, autoimmune disease and genetic factors can lead to atrophy of the gastric mucosa. Each of the four different types of gastric mucosal atrophic conditions has two subtypes; these conditions cause intraepithelial neoplasia (low grade and high grade), which eventually leads to the development of intramucosal adenocarcinoma (Fig. 1).

### Type and distribution of atrophic conditions of the gastric mucosa

**Fig. 2:**
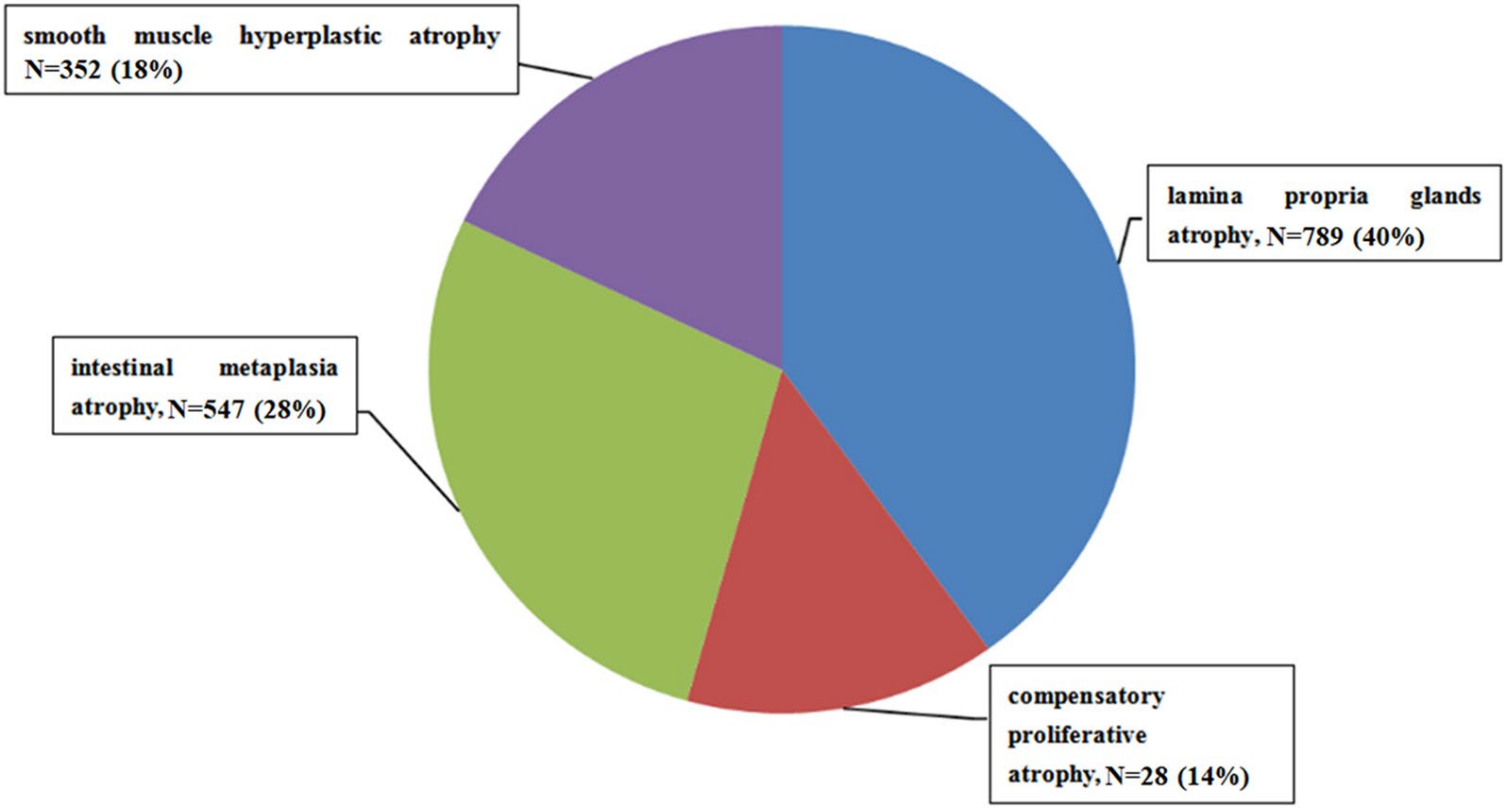
Type and distribution of atrophic lesions of the gastric mucosa

This study comprised 1969 patients with atrophic gastric mucosal lesions, including 789 patients with lamina propria atrophy (accounting for 40.07% of the total count) and 547 patients with intestinal metaplastic atrophy (accounting for 27.78% of the total count) (Fig. 2).

### Pathological stages and characteristics of atrophic conditions of the gastric mucosa

**Fig. 3:**
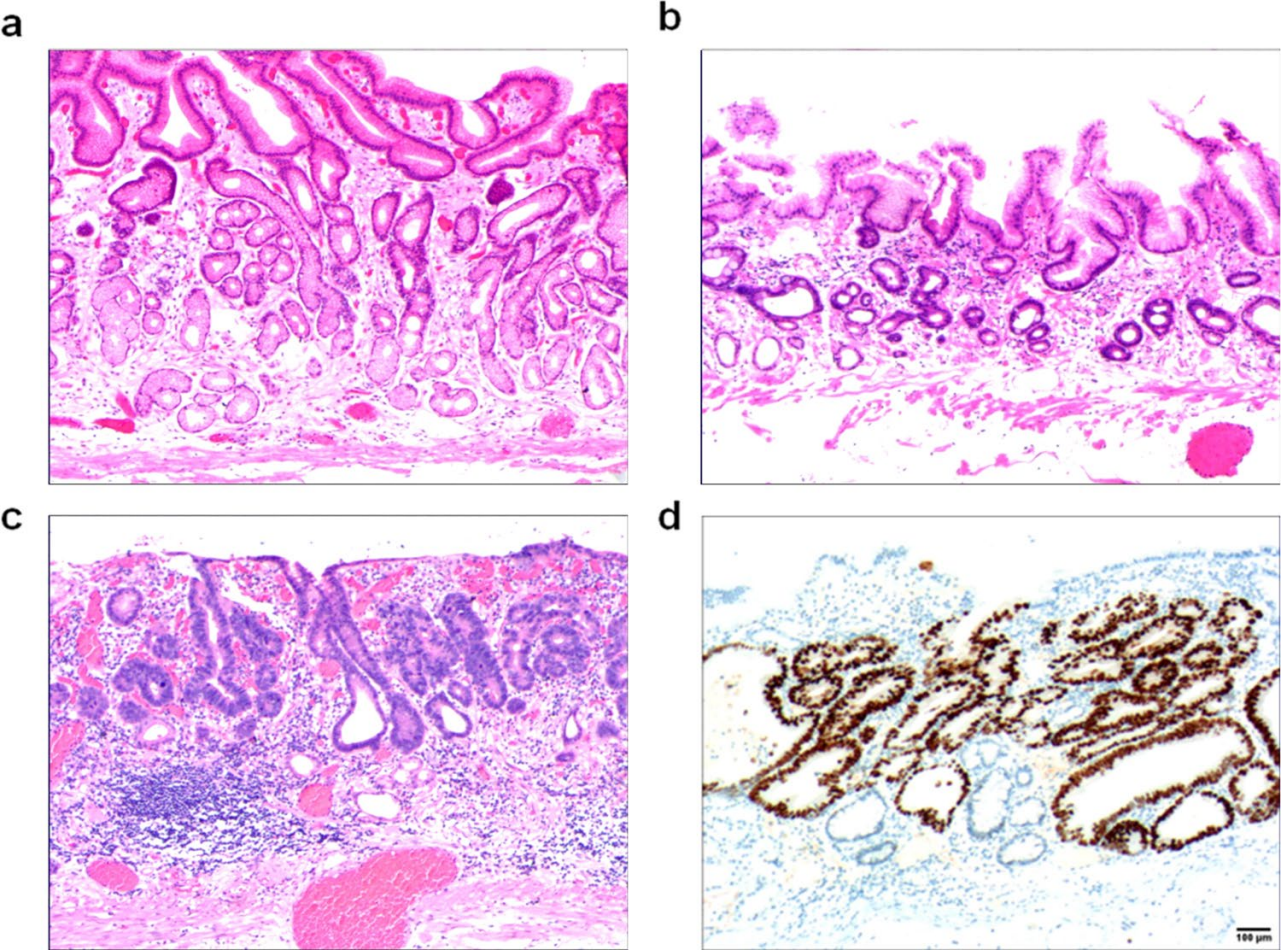
Stage I, lamina propria gland atrophy. **a** In stage IA, the lamina propria glands were mildly atrophic. The size and number of lamina propria glands were smaller (the number was reduced by 1/2 of the original glands), the glandular septa were widened and the gastric pits were shallower. Scale bar = 100 µm. **b** In stage IB, the lamina propria glands were heavily atrophic and severely reduced. The number of glands was reduced by > 1/2 of the original glands. Scale bar = 100 µm. **c** High-grade intraepithelial neoplasia with marked epithelial cell atypia, large nuclei, increased nucleoplasmic ratio, prominent nucleoli and increased mitotic figures. Scale bar = 100 µm. **d** High-grade intraepithelial neoplasia with p53 protein positive expression. Scale bar = 100 µm

**Fig. 4:**
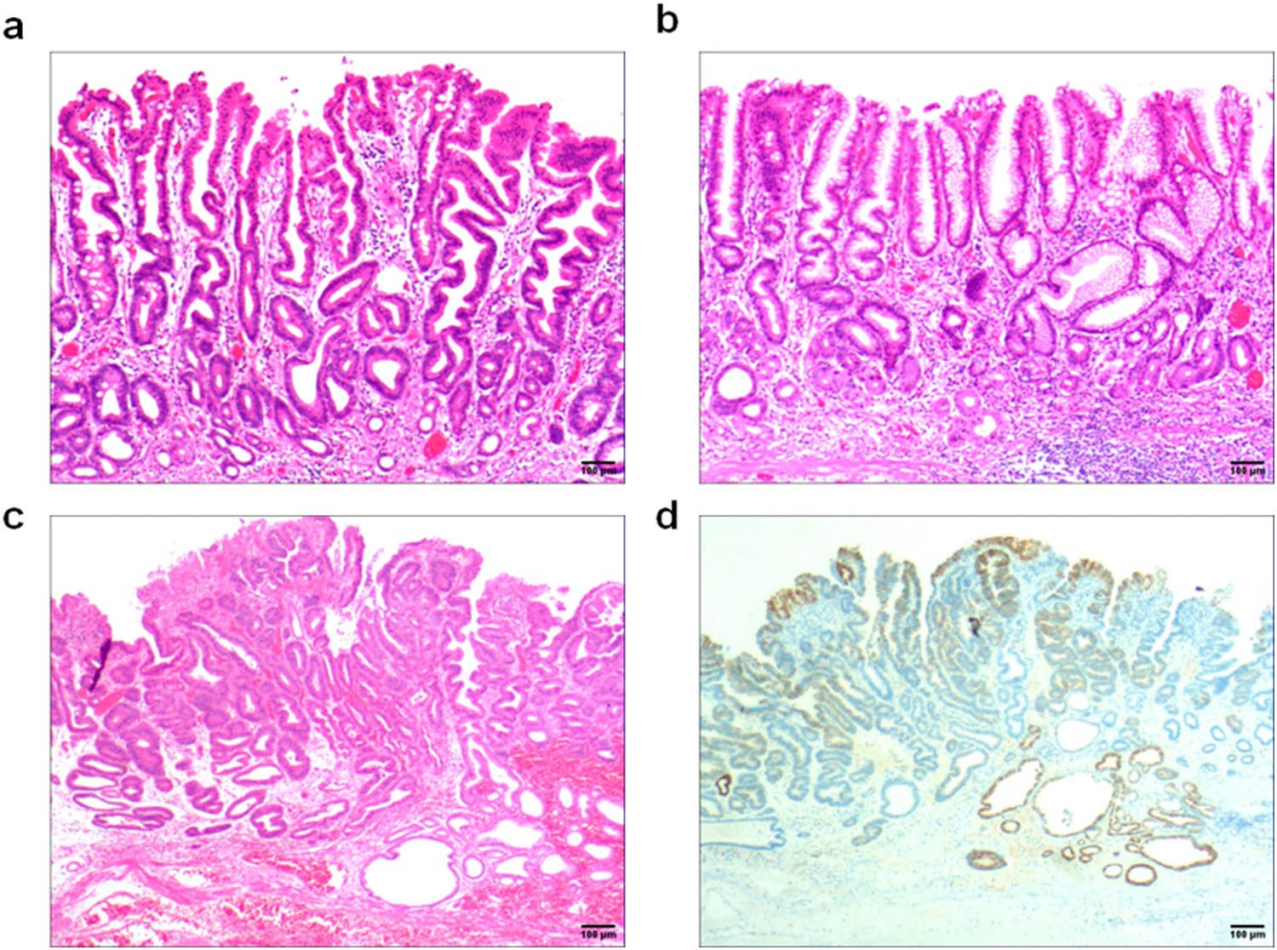
Stage II, showing compensatory proliferative atrophy. **a** In stage IIA, polar compensatory hyperplasia with (1) a decrease in lamina propria glands and (2) proliferation of stem cells and cervical mucinous cells in the proliferative zone. The surface epithelial cell proliferation showed characteristic papillary proliferation. Scale bar = 100 µm. **b** In stage IIB, the lamina propria glands were reduced, the mucosal structure was abnormal, and the polarity was disordered (mainly the surface epithelial cells appeared in the area of the fundic glands). Scale bar = 100 µm. **c** In low-grade intraepithelial neoplasia, epithelial cells showed mild to moderate atypia and longer nuclei; however, they were still polar. Scale bar = 100 µm. **d** Mucin 5AC was positively expressed. Mainly the surface epithelial cells appeared positive in the area of the fundic gland. Scale bar = 100 µm

**Fig. 5:**
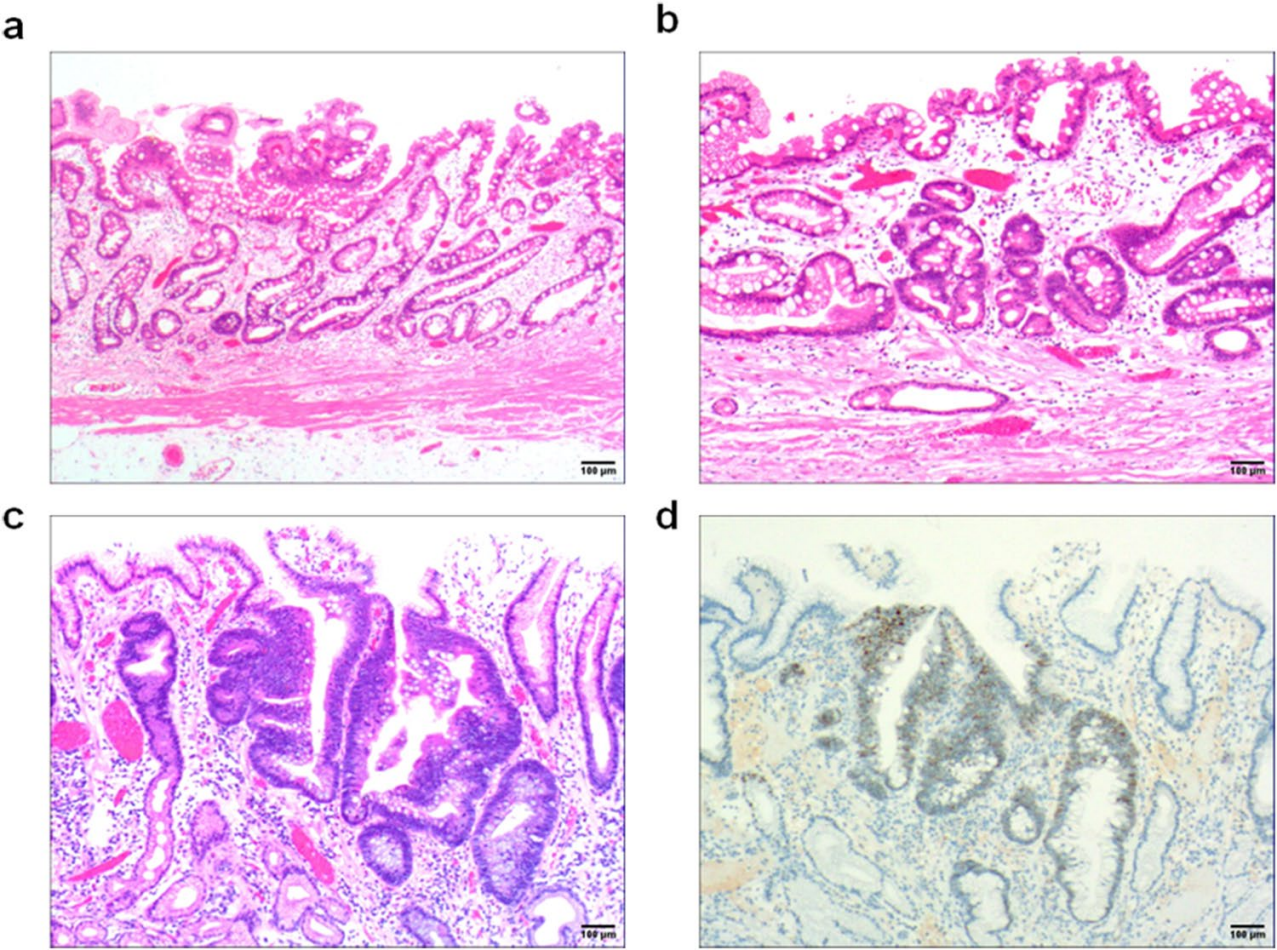
Stage III, intestinal metaplasia atrophy. **a** In stage IIIA, there was a presence of dominant intestinal metaplasia. Intestinal metaplasia cells accounted for > 30% of the entire gland, and intestinal metaplasia lesions appeared in more than three consecutive gastric areas and multiple sites in the intestinal metaplasia area. Scale bar = 100 µm. **b** In stage IIIB, intestinal metaplasia proliferated antagonistically. Concurrent with intestinal metaplasia, significant antagonistic proliferation occurred (this was mainly manifested as atypical proliferation of cells on both sides of intestinal metaplasia cells) or atypical proliferative glands appeared in the surrounding glands of intestinal metaplasia glands. Scale bar = 100 µm. **c** High-grade intraepithelial neoplasia with marked epithelial cell atypia, large nuclei, an increased nucleoplasmic ratio, prominent nucleoli, and increased mitotic figures. Scale bar = 100 µm. **d** High-grade intraepithelial neoplasia with a positive expression of p53. Scale bar = 100 µm

**Fig. 6:**
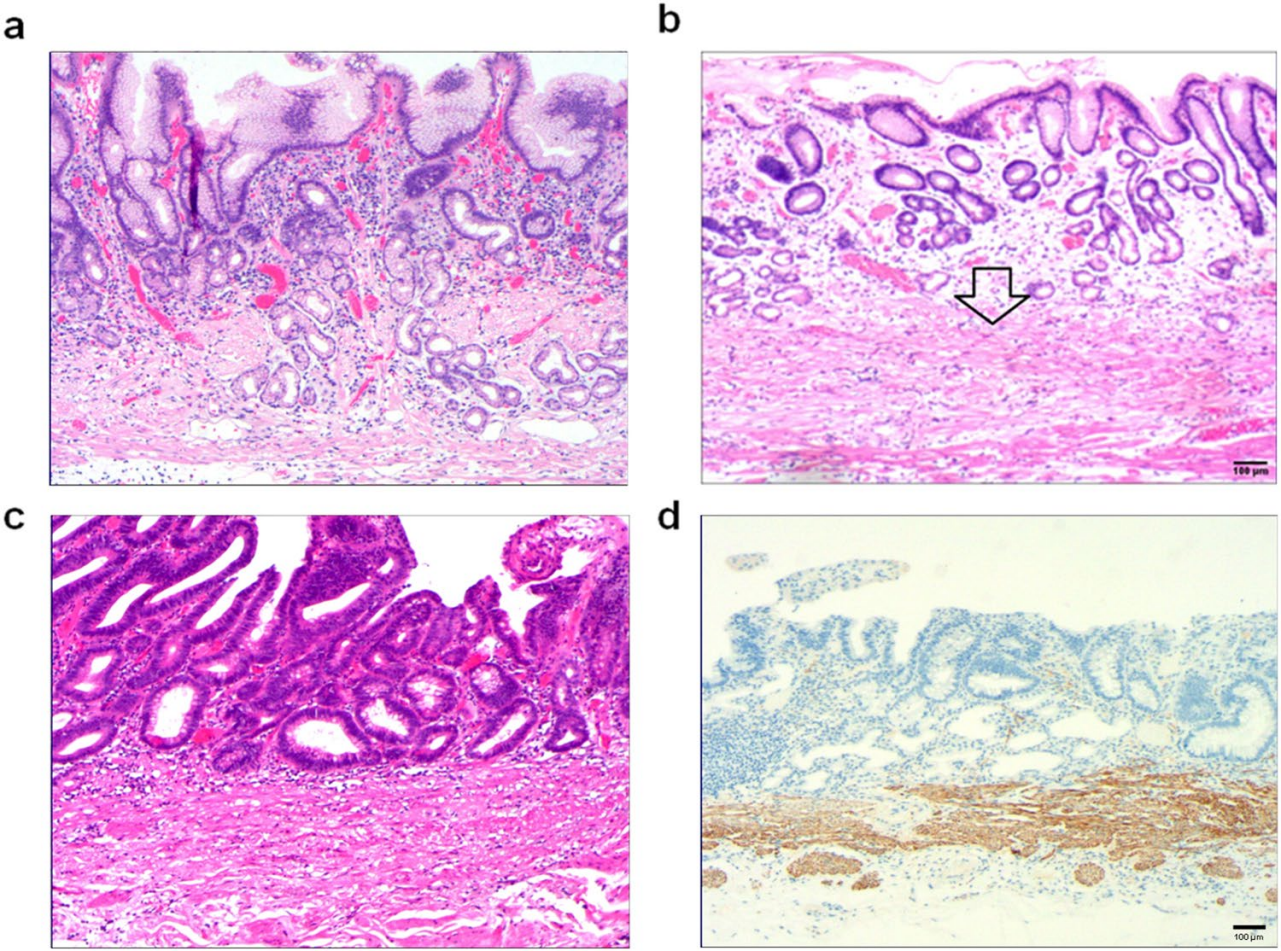
Stage IV, smooth muscle hyperplastic atrophy. **a** In stage IVA, there was smooth muscle lamellar hyperplastic atrophy. Smooth muscle fibres of varying sizes and morphologies appeared in the lamina propria; they septated to surround the lamina propria glands. Smooth muscle at the base of the lamina propria showed bundles deep into the lamina propria, separating the lamina propria glands into nests of variously sized cells. Scale bar = 100 µm. **b** In stage IVB, there was smooth muscle fibrolamellar atrophy. Significant smooth muscle proliferation in the lamina propria was seen, and in severe cases, hyperplastic smooth muscle from the mucosa to the mucosal proliferation area constituted a distinctive myofibre plate structure together with the mucosa. Scale bar = 100 µm. **c** High-grade intraepithelial neoplasia with marked epithelial cell atypia, large nuclei, an increased nucleoplasmic ratio, prominent nucleoli and increased mitotic figures. Scale bar = 100 µm. **d** Desmin was positively expressed in hyperplastic smooth muscle. Scale bar = 100 µm

Due to the differences in the aetiology and duration of gastric mucosal atrophy, the degrees of gastric mucosal damage and pathological patterns exhibited vary. The subtypes of atrophic mucosa transformation were ranked according to their supposed clinical–biological severity. (1) Stage I, lamina propria atrophy: in stage IA, mild atrophy of the lamina propria occurred; this was the beginning of atrophy of the gastric mucosa. It mainly manifested in smaller volumes in a fewer number of laminae propria glands (Fig. 3a). In stage IB, severe atrophy of the lamina propria glands occurred, with severe reduction or disappearance of the lamina propria glands (Fig. 3b) and possible development into intraepithelial neoplasia (Fig. 3c) or gastric adenocarcinoma when the lesion could not be well controlled. (2) Stage II, compensatory proliferative atrophy: in stage IIA, polar compensatory hyperplasia occurred, with a decrease in the pyloric/basal glands accompanied by a proliferation of stem cells and cervical mucous cells in the proliferative zone as well as a characteristic papillary structure of surface epithelial cell proliferation (Fig. 4a). In stage IIB, disordered compensatory hyperplasia occurred, with surface epithelial cells and cervical mucous cells in the pyloric/basal gland region (Fig. 4b) as well as a progression to intraepithelial neoplasia or gastric adenocarcinoma (Fig. 4c). (3) Stage III, intestinal metaplasia atrophy: in stage IIIA, dominant intestinal metaplasia occurred, with a high number of intestinal metaplasia cells in an extensive area (Fig. 5a). In stage IIIB, antagonistic intestinal metaplasia occurred, with the heterogeneous proliferation of cells on both sides of the intestinal metaplasia cells or heterogeneous proliferation of glands around the intestinal metaplasia glands (Fig. 5b) as well as a progression to intraepithelial neoplasia (Fig. 5c). (4) Stage IV, smooth muscle hyperplastic atrophy: in stage IVA, smooth muscle lamellar hyperplasia occurred where smooth muscles at the base of the lamina propria were bundled deep into the lamina propria, separating it into lamellar structures with one or more segments of smooth muscle hyperplasia (Fig. 6a). In stage IVB, smooth muscle fibre plate hyperplasia occurred where the hyperplastic smooth muscle extended from the mucosal muscle layer to the proliferative zone of the mucosa, forming a characteristic muscle fibre plate-like structure together with the mucosal muscle layer (0.6–0.9 mm thick) (Fig. 6b); it could also develop into intraepithelial neoplasia (Fig. 6c) or gastric adenocarcinoma. The diagnostic criteria for each stage are shown in Table 2.

**Table 2:** Relationship between age of onset of atrophic lesions of gastric mucosa and sex

Stage	*N* (%)	Sex	*P *value	Age	*P *value
		Male, female		≤ 60, > 60	
Stage I, lamina propria glands atrophy	789 (40.1)	491 (62.2), 298 (37.8)		497 (63.0), 292 (37.0)	
Stage IA, mild atrophy of lamina propriae glands	472 (24.0)	289 (61.2), 183 (38.8)	0.479	306 (64.8), 166 (35.2)	0.192
Stage IB, severe atrophy of lamina propria glands	317 (16.1)	202 (63.7), 115 (36.3)		191 (60.3), 126 (39.7)	
Stage II, compensatory proliferative atrophy	281 (14.3)	172 (61.2), 109 (38.8)		159 (56.6), 122 (43.4)	
Stage IIA, polar compensatory hyperplasia	184 (9.3)	115 (62.5), 69 (37.5)	0.541	109 (59.2), 75 (40.8)	0.216
Stage IIB, disordered compensatory hyperplasia	97 (4.9)	57 (58.8), 40 (41.2)		50 (51.5), 47 (48.5)	
Stage III, intestinal metaplasia atrophy	547 (27.8)	338 (61.8), 209 (38.2)		342 (62.5), 205 (37.5)	
Stage IIIA, dominant intestinal metaplasia	392 (19.9)	241 (61.5), 151 (38.5)	0.811	249 (63.5), 143 (36.5)	0.443
Stage IIIB, antagonistic intestinal metaplasia	155 (7.9)	97 (62.6), 58 (37.4)		93 (60.0), 62 (40.0)	
Stage IV, smooth muscle hyperplastic atrophy	352 (17.9)	226 (64.2), 126 (35.8)		206 (58.5), 146 (41.5)	
Stage IVA, smooth muscle lamellar hyperplasia	287 (14.6)	183 (63.8), 104 (36.2)	0.717	177 (61.7), 110 (38.3)	0.012
Stage IVB, smooth muscle fibre plate hyperplasia	65 (3.3)	43 (66.2), 22 (33.8)		29 (44.6), 36 (55.4)	
Total	1969	1227 (62.3), 742 (37.7)		1204 (61.1), 765 (38.9)	

### Immunohistochemical staining results

Lamina propria glandular atrophy mainly occurred with reduced or absent MUC6-positive cells and reduced MUC5AC-positive cells. Villin, CDX2 and p53 (Fig. 3d) were positively expressed in the development of intraepithelial neoplasia or intramucosal carcinoma. Compensatory proliferative atrophy mainly occurred with the extensive positive expression of MUC5AC (Fig. 4d), and MUC6 was reduced or absent. Intestinal metaplasia atrophy mainly occurred with the extensive positive expression of MUC2. Villin, CDX2 and p53 (Fig. 5d) were positively expressed in the development of intraepithelial neoplasia or intramucosal carcinoma. Smooth muscle proliferative atrophy occurred with the positive expression of SMA and DES (Fig. 6d). Villin, CDX2 and p53 were positively expressed in the development of intraepithelial neoplasia or intramucosal carcinoma. The cell proliferation index Ki-67 was an important indicator to determine cell transformation and canceration, (Table 2).

### Follow-up results

Patients presenting with little change and aggravation accounted for 85.7% (1688/1969) and 9.8% (192/1969) of the total count, respectively. Patients whose condition developed into low-grade intraepithelial neoplasia (LGIN) and high-grade intraepithelial neoplasia (HGIN) accounted for 2.8% (55/1969) and 1.1% (21/1969) of the total count, respectively. Patients whose condition developed into intramucosal carcinoma accounted for 0.7% (13/1969) (Table 3).

**Table 3:** Follow-up review results of pathological stages of atrophic lesions of gastric mucosa

Pathological stages	Number of cases	Cases (%)	Cases (%)	Cases (%)
	27	Little change weighting	LGIN HGIN	Intramucosal carcinoma
Stage I, lamina propria glands atrophy	789 (40.1)	650 (82.4); 96 (12.2)	28 (3.5); 10 (1.3)	5(0.6)
Stage IA, mild atrophy of lamina propriae glands	472 (24.0)	411 (87.1); 52 (11.0)	7 (1.5); 2 (0.4)	0(0.0)
Stage IB, severe atrophy of lamina propria glands	317 (16.1)	239 (75.4); 44 (13.9)	21 (6.6); 8 (2.5)	5 (1.6)
Stage II, compensatory proliferative atrophy	281 (14.3)	241 (85.8); 27 (9.6)	8 (2.8); 3 (1.1)	2 (0.7)
Stage IIA, polar compensatory hyperplasia	184 (9.3)	165 (89.7); 17 (9.2)	2 (1.1); 0 (0.0)	0 (0.0)
Stage IIB, disordered compensatory hyperplasia	97 (4.9)	76 (78.4); 10 (10.3)	6 (6.2); 3 (3.1)	2 (2.1)
Stage III, intestinal metaplasia atrophy	547 (27.8)	491 (89.8); 37 (6.8)	10 (1.8); 5 (0.9)	4(0.7)
Stage IIIA, dominant intestinal metaplasia	392 (1.9)	367 (93.6); 24 (6.1)	1 (0.3); 0 (0.0)	0 (0.0)
Stage IIIB, antagonistic intestinal metaplasia	155 (7.9)	124 (80.0); 13 (8.4)	9 (5.8); 5 (3.2)	4 (2.6)
Stage IV, smooth muscle hyperplastic atrophy	352 (17.9)	306 (86.9); 32 (9.1)	9 (2.6); 3 (0.9)	2(0.6)
Stage IVA, smooth muscle lamellar hyperplasia	287 (14.6)	257 (89.5); 28 (9.8))	2 (0.7); 0 (0.0)	0 (0.0)
Stage IVB, smooth muscle fibre plate hyperplasia	65 (3.3)	49 (75.4); 4 (6.2)	7 (10.8); 3 (4.6)	2 (3.1)
*χ*^2^		11.8642	0.319	0.1074
*P* value		0.007	0.956	0.990
Total	1969	1688 (85.7); 192 (9.8)	55 (2.8); 21 (1.1)	13(0.7)

*LGIN* low-grade intraepithelial neoplasia, *HGIN* high-grade intraepithelial neoplasia HGIN

## Discussion

Epidemiological and biological evidence suggests that atrophic gastritis is an important risk factor for intestinal gastric adenocarcinoma, and the significance of the histopathological assessment of ‘gastric atrophy’ and ‘atrophic gastritis’ was proposed as early as 1998 by Genta RM. Given the controversial standard definitions of both conditions, a histopathological evaluation can help improve diagnostic accuracy and estimate the risk of gastric cancer in patients [14]. In 1994, the International Symposium on Histopathology of Gastritis emphasised the importance of distinguishing between an atrophic and a non-atrophic stomach [19], as well as the types of metaplasia and non-metaplasia [16]. A previous study found that the determination of the RAS oncogene product p21 and DNA content was important in the diagnosis of atrophic gastritis. Subsequently, the significance of the simple, hyperplastic, intestinal metaplasia and atypia types of atrophic gastritis was proposed [20–22]. The gastric mucosa is composed of three layers: (1) the epithelium; (2) the lamina propria; and (3) the mucosal muscle layer. The normal thickness of the gastric mucosa in adults is 0.7–1.1 mm in the cardia region, 0.8–1.2 mm in the fundus region, 0.9–1.3 mm in the gastric body region and 1.0–1.4 mm in the antrum region [23]. In this study, it was found that when the epithelium of the gastric mucosa was affected by infection, chemical stimulation, immune factors or genetic factors, it led to atrophy of the gastric mucosal epithelium and glands, thinning of the mucosa, reduction in the number of glands, intestinal epithelial hyperplasia and proliferation of smooth muscle fibres. In addition, the proliferation and dysplasia of gastric epithelial cells, characterised by varying degrees of cellular and structural atypia, lead to neoplastic hyperplasia in nature; this is collectively known as gastric mucosal atrophic lesions. The gastric mucosal atrophy’s start from the proliferative zone was confirmed by histopathological characteristics and follow-up analysis. It was also found that gastric mucosal atrophy was not caused by chronic gastritis and that gastric mucosal atrophy was not always accompanied by gastritis. Therefore, the nomenclature of atrophic lesions of the gastric mucosa was proposed in this study.

In the normal histology of the gastric mucosa, the bottom of each gastric pit is connected to three to five glands called gastric units, which are the basic structural units of the gastric mucosa. The gastric unit is composed of the gastric pits and the gastric glands, which are subdivided into canyons, glandular necks and bases. In the deep part of the gastric pits, the upper part of the canyons and the neck of the gland are called the proliferative zone. From the top of the fundic glands to the deep part of the gastric pits, the cells are small and low columnar (mainly proliferating stem cells). Some migrate upwards and differentiate into surface mucinous cells, while some stay locally or migrate downwards and differentiate into other fundic glandular cells [24–27]. Through the observation of 1969 patients with atrophic lesions of the gastric mucosa in this study, it was found that gastric mucosal atrophy occurred when the epithelium of the gastric mucosa was affected by infection, chemical stimulation, immune factors and genetic factors, resulting in the failure of stem cells in the proliferative zone of the gastric mucosa to differentiate and migrate normally. The formation of gastric mucosal atrophy can cause structural disorder or absence of the proliferative zone, leading to insufficient downward migration, which morphologically appears as glandular atrophy of the lamina propria. Conversely, the excess proliferation of the proliferative zone leads to the transformation of downward migrating cells, which morphologically appear as intraepithelial neoplasia. A histopathological staging was developed based on the process of occurrence and development of (1) glandular atrophy of the gastric mucosa and (2) the histomorphological features from benign to malignant. The lamina propria glandular atrophy type mainly emphasises the reduction or complete loss of lamina propria glands, which is also an early stage of atrophic gastric mucosal lesions. It progresses gradually from light atrophy of the lamina propria glands to heavy atrophy of the lamina propria glands, developing into intramucosal carcinoma in 1.6% at a 1- to 4-year follow-up period. The compensatory hyperplastic atrophy of the gastric mucosa mainly emphasises the reduction of glands in the lamina propria and the proliferation of stem cells and cervical mucous cells in the proliferative zone, forming a characteristic papillary proliferation of surface epithelial cells. Gastric mucosal intestinal metaplastic atrophy mainly emphasises the progression from initially protective to antagonistic and finally to intramucosal gastric cancer. Gastric mucosal smooth muscle proliferative atrophy is a late-stage condition of gastric mucosal atrophic lesions. The pathological staging proposed in this study was mainly based on the histological structure and cytomorphological characteristics of gastric mucosal atrophy as well as the hypothesis of malignant transformation of cells during the occurrence and development of the lesion. Using immunohistochemical analysis, it was found that villin, CDX2 and p53 were positively expressed in the development of intraepithelial neoplasia or intramucosal carcinoma; IHC staining analysis showed that MUC6 and MUC5AC were reduced. Writing this histopathological grading in the clinical pathology test report will be of great significance in guiding clinicians for the precise treatment and tracking of malignant cell transformation as well as in controlling the development of gastric cancer.

Gastric cancer is one of the most common types of cancer worldwide, and gastric mucosal atrophy is clearly associated with the development of the disease [7, 28]. The degree of gastric mucosal atrophy is associated with the risk of carcinogenesis, and the endoscopic assessment of gastric mucosal atrophy is a simple and very important tool for identifying people at high risk of gastric cancer [29]. The percentage of the included patients was (1) 85.7% patients with atrophic gastric mucosal conditions who had no obvious histological or cellular changes; (2) 9.8% patients with progressive worsening; and (3) 1.1% and 0.7% patients with a progression to high-grade intraepithelial neoplasia and intramucosal carcinoma, respectively. This was found through follow-ups via gastric mucosal biopsy for 1–4 years. It is only a matter of time before the development of gastric mucosal atrophic lesions leads to gastric adenocarcinoma. Therefore, the follow-up of the malignant cell transformation of gastric mucosal atrophic lesions should be emphasised, and it is essential to establish a follow-up review biopsy mechanism for gastric mucosal atrophic lesions and to conduct follow-ups at intervals of every 3, 6 and 12 months according to histopathological stages to control the occurrence of gastric cancer.

In summary, this study proposed a nomenclature of atrophic lesions of the gastric mucosa and found that gastric mucosal atrophy starts from the proliferative zone. The histopathological staging of gastric mucosal atrophic conditions was developed based on the histological structure and cytomorphological features of gastric mucosal atrophy and the pattern of malignant transformation of cells during the development and progression of the lesions. Although there have been no guidelines yet, the pathological types of gastric mucosal atrophic lesions had been written in the clinical pathology test report, guiding clinicians in the precise treatment and follow-up of malignant cell transformation, which was important for controlling the occurrence and development of gastric cancer. The proliferative zone of the gastric mucosa contained a variety of low-differentiated cells with different differentiation directions, and it was still unclear what kind of cells in the proliferative zone had been responsible for gastric mucosal atrophy. In addition, further studies on molecular biology and observation of a large number of patients and follow-ups are required.

## Data Availability

All data generated or analysed during this study are included in this published article.

## References

[1] Draşovean SC, Boeriu AM, Akabah PS (2018). Optical biopsy strategy for the assessment of atrophic gastritis, intestinal metaplasia, and dysplasia. Rom J Morphol Embryol.

[2] Pokhrel N, Khanal B, Rai K (2019). Application of PCR and microscopy to detect helicobacter pylori in gastric biopsy specimen among acid peptic disorders at tertiary care centre in Eastern Nepal. Can J Infect Dis Med Microbiol.

[3] Urabe M, Ushiku T, Shinozaki-Ushiku A (2018). Adenocarcinoma of the esophagogastric junction and its background mucosal pathology: a comparative analysis according to Siewert classification in a Japanese cohort. Cancer Med.

[4] Correa P (1988). Chronic gastritis: a clinico-pathological classification. Am J Gastroenterol.

[5] Genta RM (1996). Recognizing atrophy: another step toward a classification of gastritis. Am J Surg Pathol.

[6] Massironi S, Zilli A, Elvevi A (2019). The changing face of chronic autoimmune atrophic gastritis: an updated comprehensive perspective. Autoimmun Rev.

[7] Akbari M, Tabrizi R, Kardeh S (2019). Gastric cancer in patients with gastric atrophy and intestinal metaplasia: a systematic review and meta-analysis. PLoS ONE.

[8] Yoon JH, Lee YS, Kim O (2019). NKX6.3 protects against gastric mucosal atrophy by downregulating β-amyloid production. World J Gastroenterol.

[9] Offerhaus GJ, Price AB, Haot J (1999). Observer agreement on the grading of gastric atrophy. Histopathology.

[10] Kim HJ, Kim N, Yun CY (2019). The clinical meaning of the "indefinite for atrophy" lesions within gastric mucosa biopsy specimens in a region with a high prevalence of gastric cancer. Helicobacter.

[11] Bockerstett KA, Osaki LH, Petersen CP (2018). Interleukin-17A promotes parietal cell atrophy by inducing apoptosis. Cell Mol Gastroenterol Hepatol.

[12] Sipponen P (2021). Prevalence rates of heathy stomach mucosa, chronic non-atrophic and atrophic gastritis in endoscopic biopsies in adults born in Finland in 1890–1977. Scand J Gastroenterol.

[13] El-Zimaity H (2008). Gastritis and gastric atrophy. Curr Opin Gastroenterol.

[14] Genta RM (1998). Review article: gastric atrophy and atrophic gastritis–nebulous concepts in search of a definition. Aliment Pharmacol Ther.

[15] Matysiak-Budnik T, Camargo MC, Piazuelo MB (2020). Recent guidelines on the management of patients with gastric atrophy: common points and controversies. Dig Dis Sci.

[16] Rugge M, Correa P, Dixon MF (2002). Gastric mucosal atrophy: interobserver consistency using new criteria for classification and grading. Aliment Pharmacol Ther.

[17] Crafa P, Russo M, Miraglia C (2018). From Sidney to OLGA: an overview of atrophic gastritis. Acta Biomed.

[18] Zhang ZS, Deng WY, Huang SL, Yang BF, Zhu FH, Jiang B, Wang SN, Wang YK (2022). Clinicopathological characteristics of signet-ring cell carcinoma derived from gastric fovelar epithelium. J Dig Dis.

[19] Dixon MF, Genta RM, Yardley JH (1996). Classification and grading of gastritis. The updated sydney system. International workshop on the histopathology of gastritis, Houston 1994. Am J Surg Pathol.

[20] Wang YK, Ma NX (1997). Expression of four gene proteins such as P21 in atrophic gastritis and determination of DNA content. Chin Med J.

[21] Wang YK, Ma NX (1998). Study on the content of ras oncogene product P21 and DNA in atrophic gastritis. Chin J Cancer.

[22] Wang YK, Shen L, Yun T (2021). Histopathological classification and follow-up analysis of chronic atrophic gastritis. World J Clin Cases.

[23] Wang YK. Gastric nonneoplastic lesions [M]//Chun-fang Gao,Yang-kun Wang, Editor-in-Chief. Digestive system disease diagnosis and treatment.Beijing:People's Military Medical Publishing House. 2016;581–687.

[24] Russi S, Calice G, Ruggieri V (2019). Gastric normal adjacent mucosa versus healthy and cancer tissues: distinctive transcriptomic profiles and biological features. Cancers (Basel).

[25] Souza SM, Valiente AEF, Sá KM (2019). Immunoexpression of LGR4 and beta-catenin in gastric cancer and normal gastric mucosa. Asian Pac J Cancer Prev.

[26] Tarnawski AS, Ahluwalia A (2018). Increased susceptibility of aging gastric mucosa to injury and delayed healing: clinical implications. World J Gastroenterol.

[27] Heidari Z, Mahmoudzadeh-Sagheb H, Narouei M (2017). Quantitative changes of gastric mucosa during carcinogenesis using stereological methods. J BUON.

[28] Song JH, Kim SG, Jin EH (2017). Risk factors for gastric tumorigenesis in underlying gastric mucosal atrophy. Gut Liver.

[29] Masuyama H, Yoshitake N, Sasai T (2015). Relationship between the degree of endoscopic atrophy of the gastric mucosa and carcinogenic risk. Digestion.

